# ASSOCIATIONS BETWEEN SLEEP HYGIENE INDEX SCORE AND WHITE MATTER MICROSTRUCTURE

**DOI:** 10.1093/geroni/igae098.3516

**Published:** 2024-12-31

**Authors:** Lisa Weimar, Meina Zhang, Vincent Magnotta, Eric Axelson, Josh Cochran, Lauren Hopkins, Chooza Moon

**Affiliations:** University of Iowa, Iowa City, Iowa, United States; University of Iowa, Iowa City, Iowa, United States; University of Iowa, Iowa City, Iowa, United States; University of Iowa College of Medicine, Iowa City, Iowa, United States; University of Iowa, Iowa City, Iowa, United States; University of Iowa, Iowa City, Iowa, United States; University of Iowa, Iowa City, Iowa, United States

## Abstract

Sleep complaints are common among older adults. Sleep hygiene refers to practices that promote adequate sleep and may represent an interventional target for improving sleep quality. Individuals with Alzheimer’s disease often exhibit white matter (WM) microstructure degenerations observable through diffusion tensor imaging (DTI). As people age, poor sleep quality can increase the risk of developing Alzheimer’s disease and damage WM microstructures. However, few studies have investigated the relationship between sleep hygiene and the structural integrity of WM among older adults. Data was collected from 49 older adults (51% female) with mean age 70.4 years (SD = 6.50). Diffusion-weighted MRI sequences were acquired using a 3.0T scanner. Fractional anisotropy (FA) scalar maps were generated through FSL-DTIFIT, and the Johns Hopkins University-ICBM white matter atlas was utilized to assess FA values within WM tracts. Participants completed the Sleep Hygiene Index (SHI), which is a self-reported instrument. In this preliminary analysis, poorer sleep hygiene was associated with lower FA values in the bilateral cerebral peduncles and posterior limb internal capsules, right anterior limb internal capsule, and left uncinate fasciculus compared to those who have better sleep hygiene. This preliminary data suggests that among this small, homogenous sample poor sleep hygiene is associated with reduced WM integrity. Future studies should include larger samples, longitudinal designs, comprehensive sleep measures, and multimodal imaging methods to confirm these findings and identify the mechanisms underlying these associations.

